# Engineering functional BMP-2 expressing teratoma-derived fibroblasts for enhancing osteogenesis

**DOI:** 10.1038/s41598-018-32946-6

**Published:** 2018-10-01

**Authors:** Yoon Young Go, Ji Yeon Mun, Sung-Won Chae, Shin Hye Kim, Hoseok Song, Jae-Jun Song

**Affiliations:** 1 Department of Otorhinolaryngology-Head and Neck Surgery, Korea University Guro Hospital, Seoul, 08308 Republic of Korea; 2 Department of Biomedical Sciences, Korea University College of Medicine, Seoul, 02841 Republic of Korea

## Abstract

Bone morphogenetic protein 2 (BMP-2) is considered an effective growth factor for bone formation, and is used for making osteo-inductive scaffolds, but the related clinical investigations have shown low success rates. In this study, we genetically manipulated teratoma-derived fibroblast (TDF) cells by simultaneous introduction of *BMP-2* and *herpes simplex virus-thymidine kinase (HSV-tk)* encoding genes. Self-production of BMP-2 in TDF cells strongly enhanced the alkaline phosphatase (ALP) activity, calcium content, and elevated the mRNA expression of osteogenic marker genes during *in vitro* osteogenesis. The bone formation volume was also remarkably enhanced in calvarial and femoral critical-size defect models. Ganciclovir (GCV) treatment induced apoptosis in TDF cells co-expressing HSV-tk and BMP-2, implying that HSV-tk suicide gene can modulate the side-effects of stem cell therapy, e.g., development of uncontrollable teratoma and tumor formation. Altogether, our findings revealed a safe and highly efficient technique with potential therapeutic applications for bone regeneration.

## Introduction

Mesenchymal stem cells (MSCs) have been considered a promising cell source in the field of regenerative medicine because they are easy to harvest and can differentiate into various mesodermal tissues, such as fat, bone, and cartilage^1^. A large number of cells are needed for successful cell-based therapies, requiring extensive *in vitro* cell culturing^2^. However, it is difficult to obtain a stable phenotype of MSCs, as they readily lose their properties with cellular senescence during long culture periods^3^. Therefore, cell-based strategies using MSCs have not been widely applied in clinical studies.

Teratoma is a benign tumor composed of three germ layers: ectoderm, mesoderm, and endoderm, with disorganized mixture of tissues. Teratoma formation is considered a standard method to determine the differentiation potential of pluripotent embryonic stem cells (ESCs) and induced-pluripotent stem cells (iPSCs) into all tissue types^4,5^. The ability of ESCs and iPSCs to differentiate into all tissue types results in the formation of teratoma in immune-compromised mice. However, it is unknown whether teratoma-derived fibroblasts (TDFs) have the potential for use in bone regeneration as a cell source, under cell-mediated regenerative medicine.

Compared with MSCs, one of the major advantages of TDFs for bone regeneration is their rapid growth and able to easily manipulate a gene of interest for gene function analysis. TDFs can be isolated in large amounts from teratoma and stably maintained under *in vitro* culture conditions. However, in the context of *in vivo* growth for clinical use, a comparative characterization of TDFs and MSCs has not been done.

In this study, we isolated fibroblasts from a teratoma, which was generated by the transplantation of human ESCs (H9) into immune-deficient mice. The isolated fibroblasts showed a potential ability similar to that of MSCs to differentiate into osteoblasts. In addition, we introduced the Bone morphogenetic protein 2 (BMP2) and herpes simplex virus thymidine kinase (HSV-tk) encoding genes into the TDFs, generating a functional TDF cell line that remarkably induced bone growth and regeneration under *in vivo* conditions.

There might be a concern about the emergence of cancer cell-like features in the remaining TDF population after the *in vivo* stimulation of bone regeneration. Several previous reports have showed that the re-injection of TDFs did not re-establish teratomas in the mice with severe combined immunodeficiency (SCID)^6^. Moreover, the BMP-2 and HSV-tk genes co-expressed on TDF (TDF BMP2/HSV-tk) cells in this study exclude this possibility, due to the presence of HSV-tk/ganciclovir (GCV) system. The treatment of TDF BMP2/HSV-tk cells with GCV, which allows selective elimination of HSV-tk-expressing cells by apoptosis, successfully removed over 80% of the cells in our study. These functional TDFs could be eliminated by GCV treatment after bone formation in the affected region.

## Results

### TDFs have multi-lineage potential of differentiating to mesenchymal tissues

**Figure 1:**
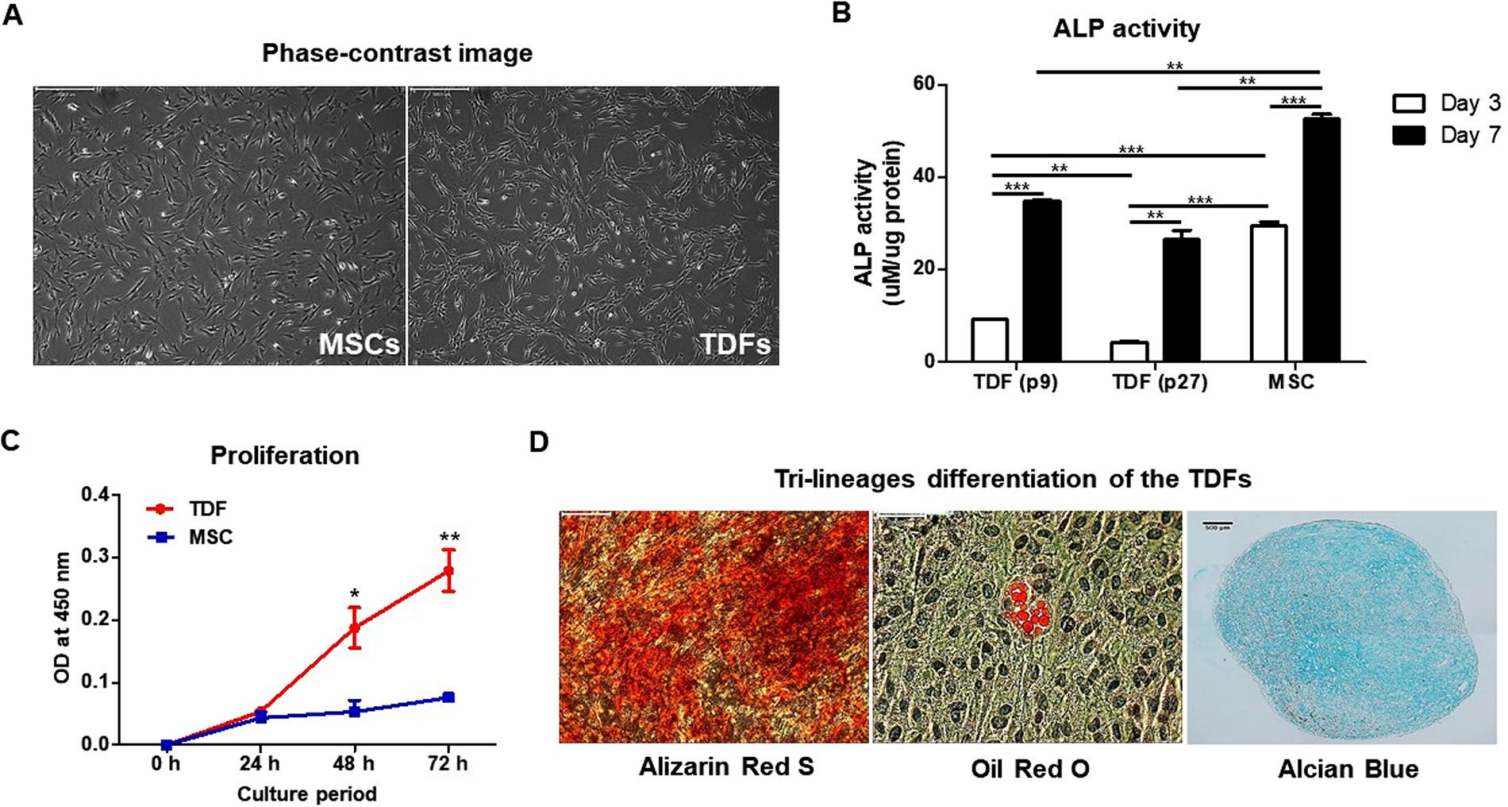
Comparison of TDFs with MSCs. (**A**) Morphology of TDFs and MSCs visualized by light microscopy. The scale bar represents 500 μm. (**B**) TDFs (p9: passage 9, and p27: passage 27) and MSCs were cultured in osteogenic induction medium and assayed for ALP activity at days 3 and 7. (**C**) TDFs (passage 22) and MSCs were seeded in a 96-well plate and then cultured in each growth medium. Cell viability was determined by CCK-8 at 24, 48, and 72 h. (**D**) TDFs differentiated into tri-lineages in each differentiation medium and osteogenesis (left), adipogenesis (middle), and chondrogenesis (right) were evaluated at day 21. Scale bars, 500 μm. Data were represented as mean ± SD; *p < 0.05, **p < 0.01, and ***p < 0.001 compared with the corresponding control.

We first observed the morphology of the two kinds of cells. The phase-contrast image showed that TDFs resembled the morphology of MSCs (Fig. 1A). Both early (passage 9) and late passage (passage 27) TDFs and MSCs were cultured in osteogenic induction medium, and their ALP activity was determined at days 3 and 7. A higher ALP activity was detected in the MSCs cultured with osteogenic induction medium in compare to both early and late passage TDFs at day 7. ALP activity of MSCs at day 3 was significantly higher than that of TDFs, showing the great capacity of MSCs as osteoblasts. Similar to the early passage TDFs, late passage TDFs also showed induction of ALP activity at day 7 under the same *in vitro* osteogenic conditions, suggesting that even the late passage TDFs are capable of osteogenic differentiation into osteoblast-like cells (Fig. 1B). To compare the proliferation between MSCs and TDFs, both cells were cultured in growth medium and the cell numbers were determined from 24 h onwards. The TDFs gradually increased in cell number, whereas MSCs sustained the cell growth with no significant increase in proliferation rate. The TDF cell numbers increased 4–5 folds, compared with the MSCs, during the period from 48 h to 72 h (Fig. 1C). We then investigated whether TDFs have the tri-lineage differentiation capacity like MSCs. For osteogenesis, TDFs were cultured in osteogenic induction medium and the mineralization of extracellular matrix was determined by Alizarin Red S staining on day 21. For adipogenesis, Oil Red O staining was used to examine the small lipid droplets in the cytoplasm of differentiated TDFs on day 14. The micromass cultures of TDFs for 3 weeks demonstrated proteoglycan-rich extracellular matrix, as revealed by Alcian blue staining (Fig. 1D). These results demonstrated that TDFs have some distinct similarities with MSCs, and can differentiate into multiple tissues such as bone, fat, and cartilage.

### Osteogenic differentiation of TDFs

Next, we further confirmed the osteogenic capacity of TDFs for tri-lineages mesenchymal differentiation. The ALP activity was evaluated in TDFs at days 3 and 7, and the results showed that the ALP activity increased significantly in the TDFs cultured in osteogenic induction medium compared with those cultured in the growth medium (Supplementary Fig. S1A). Analysis of the TDF gene expression profile at 21 days of culture showed considerable up-regulation of various osteogenic genes (*Runx2*, *OCN*, and *OPN*). The expression levels of *Runx2* and *OCN* genes were elevated at day 7, while the late osteogenic marker gene, *OPN* was up-regulated at day 21 (Supplementary Fig. S1B). The calcification of TDFs was determined by calcium assay and Alizarin Red S staining after culturing in osteogenic induction medium. A higher calcium content and mineralization formation were observed in osteogenic medium at day 21, compared with the TDFs cultured in growth medium (Supplementary Fig. S1C and D). These results clearly showed the osteogenic potential of TDFs as *in vitro* pilot models.

### Comparison of osteogenic potential of TDFs with osteoblast-like cells

**Figure 2:**
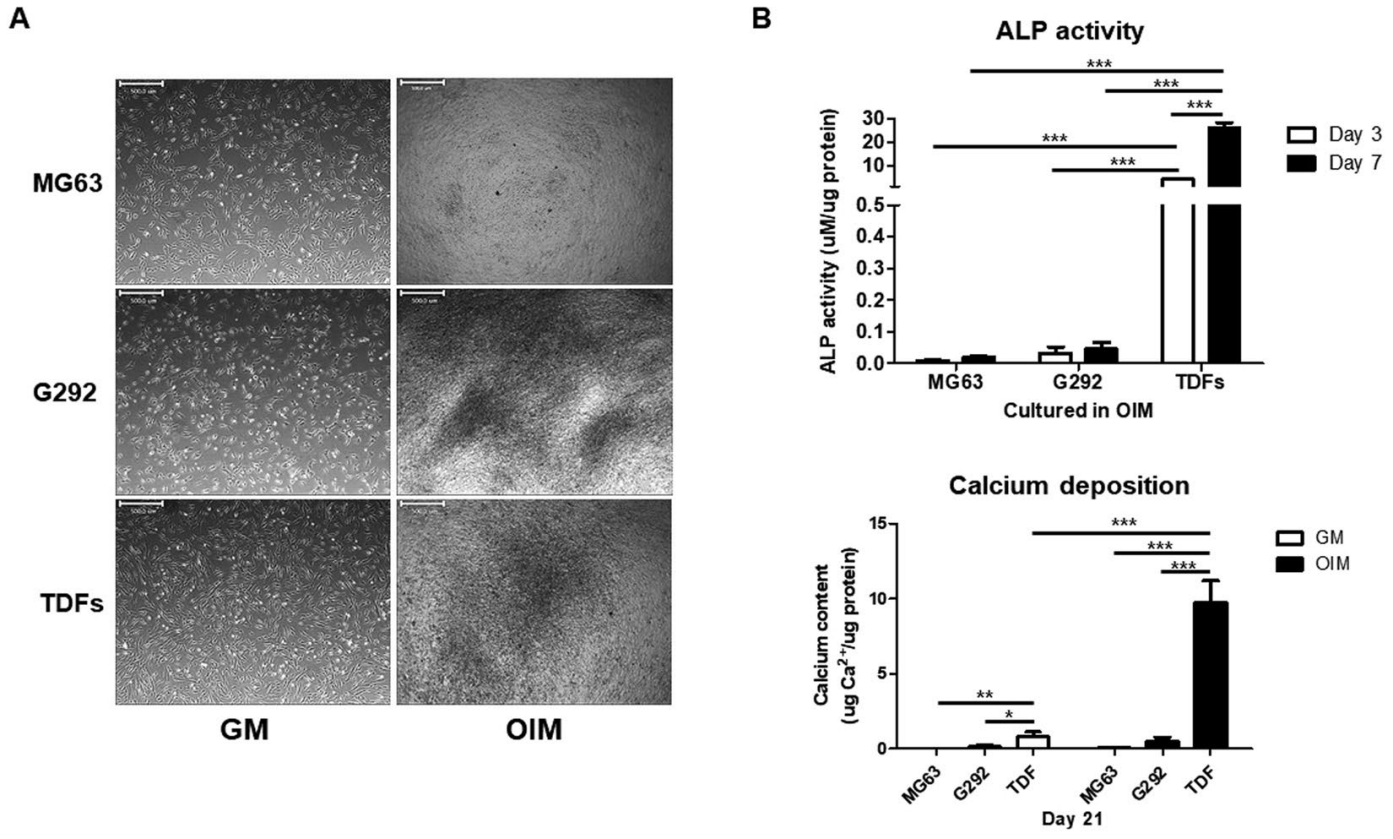
TDFs induce more osteogenic differentiation than human osteoblast-like cells. (**A**) Three human osteoblast-like cells (MG-63 and G292) and TDFs were cultured in osteogenic induction medium. Typical morphological changes after osteogenic differentiation for 7 days shown are here. The scale bar represents 500 μm. (**B**) The activity of ALP at days 3 and 7 in MG-63, G292, and TDFs were assessed and normalized to the protein content. **(C**) At day 12, the calcium contents of each cell line were determined and normalized to the protein concentration. Data were represented as mean ± SD; *p < 0.05, **p < 0.01, and ***p < 0.001 compared with the corresponding control.

We compared four osteoblast-like cells with TDFs to assess the suitability of TDFs as *in vitro* osteoblast cell models for bone research. *In vitro* ALP activity and calcium content were determined in three human osteoblast-like cells (G292 and MG63) and TDFs at the indicated day during osteogenic differentiation. The representative images of each cell type are shown in Fig. 2A. Cells cultured in the osteogenic induction medium for 7 days showed osteogenesis-related morphological changes in all cell types, as per our previous report. TDFs induced 2-fold higher ALP levels at day 7 compared with MG63 and G292cells (Fig. 2B). On day 21, calcium assay was performed to determine the calcium content of each cell line. Calcium deposition in the extracellular matrix of TDFs was also significantly higher compared with the other immortalized osteoblast-like cells (Fig. 2B), confirming that TDFs have a greater ability of osteogenesis compared with osteoblast-like cell lines.

### TDF BMP2/HSV-tk promote osteogenesis by secretion of BMP2

**Figure 3:**
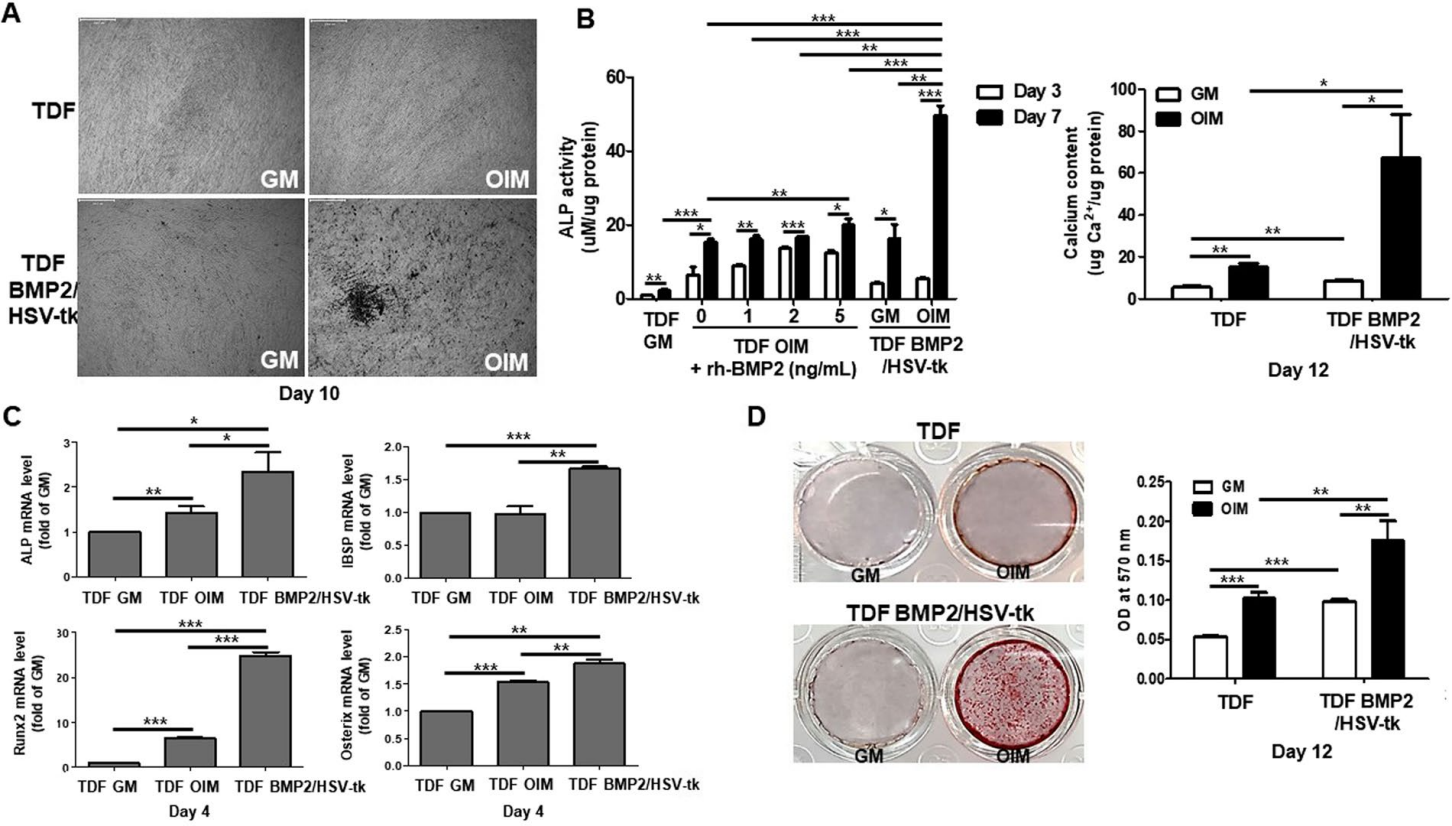
Osteogenic efficacy of TDF BMP2/HSV-tk. (**A**) TDF vehicle control and TDF BMP2/HSV-tk cells were cultured in osteogenic induction medium for 10 days. Morphological changes in TDF BMP2/HSV-tk were examined by light microscopy. The scale bar represents 500 μm. (**B**) ALP activity was measured in the TDFs treated with or without rh-BMP2 (1, 2, and 5 ng/mL), at days 3 and 7 after the *in vitro* osteogenesis in osteogenic induction medium. *In vitro* ALP activity of TDF BMP2/HSV-tk was also determined at the same time and compared with the values for ALP enzyme. Calcium contents of TDF vehicle control and TDF BMP2/HSV-tk were evaluated at day 12 after culturing in osteogenic induction medium. (**C**) The mRNA levels of osteogenic-related marker genes (*ALP*, *IBSP*, *Runx2*, and *Osterix*) were measured in TDF and TDF BMP2/HSV-tk treated with either growth medium or osteogenic induction medium. (**D**) TDF and TDF BMP2/HSV-tk were cultured in osteogenic induction medium or growth medium for 12 days. Mineralization was determined by Alizarin Red S staining. TDF vehicle control was stably introduced in only the vehicle plasmid without any encoding genes. Error bars indicated mean ± SD; *p < 0.05, **p < 0.01, and ***p < 0.001compared with the corresponding control.

We generated the functional TDF cells which co-expresses BMP2 and HSV-tk encoding genes in TDF cells (Supplementary Fig. S2A and B). To examine the osteogenic differentiation of TDF BMP2/HSV-tk, we determined the morphological changes (Fig. 3A) as well as the ALP activity and calcium deposition of cells cultured in osteogenic induction medium for 12 days. TDF BMP2/HSV-tk remarkably enhanced the ALP activity compared with the vehicle control TDF and recombinant human BMP2 (rh-BMP2) treatment groups. TDF cultured in the presence of rh-BMP2 (1, 2, and 5 ng/mL) showed no significant ALP activity at day 7, implying that self-production of BMP2 in TDF cells is more effective in promoting osteogenic differentiation compared with the extracellular treatment of rh-BMP2 (Fig. 3B). In addition, the total calcium content in TDF BMP2/HSV-tk was significantly higher in osteogenic induction medium than in the vehicle control TDFs at day 12 (Fig. 3B). We also observed an increased expression of osteogenic marker genes, including *ALP*, *IBSP*, *Runx2*, and *Osterix* coding genes, in TDF BMP2/HSV-tk cultured under osteogenic conditions (Fig. 3C). The proliferation rate of TDF and TDF-BMP2/HSVtk was similarly higher than MSCs, indicating that self-production of BMP-2 in TDF cells are actively involved enhancing the ALP activity and calcium accumulation, not the differences of proliferation rate (Supplementary Fig. S2C). Consistent with this, the staining result with Alizarin Red S also showed a higher induction of mineralization in TDF BMP2/HSV-tk than in the vehicle control TDF (Fig. 3D). The expression levels of BMP2 mRNA and protein in the middle stage of osteogenic differentiation were determined. The expression levels of *BMP2* gene and the secreted BMP2 during osteogenesis were increased in TDF BMP2/HSV-tk (Supplementary Fig. S3A and B). In addition, we investigated the involvement of BMP2 RIB in secreted BMP2 driven osteogenic differentiation of TDF BMP2/HSV-tk (Supplementary Fig. S3C). Taken together, TDF BMP2/HSV-tk significantly promoted osteogenic differentiation through the self -produced BMP2, but the rh-BMP2 treated TDF controls did not.

### TDF BMP2/HSV-tk are eliminated by GCV treatment

**Figure 4:**
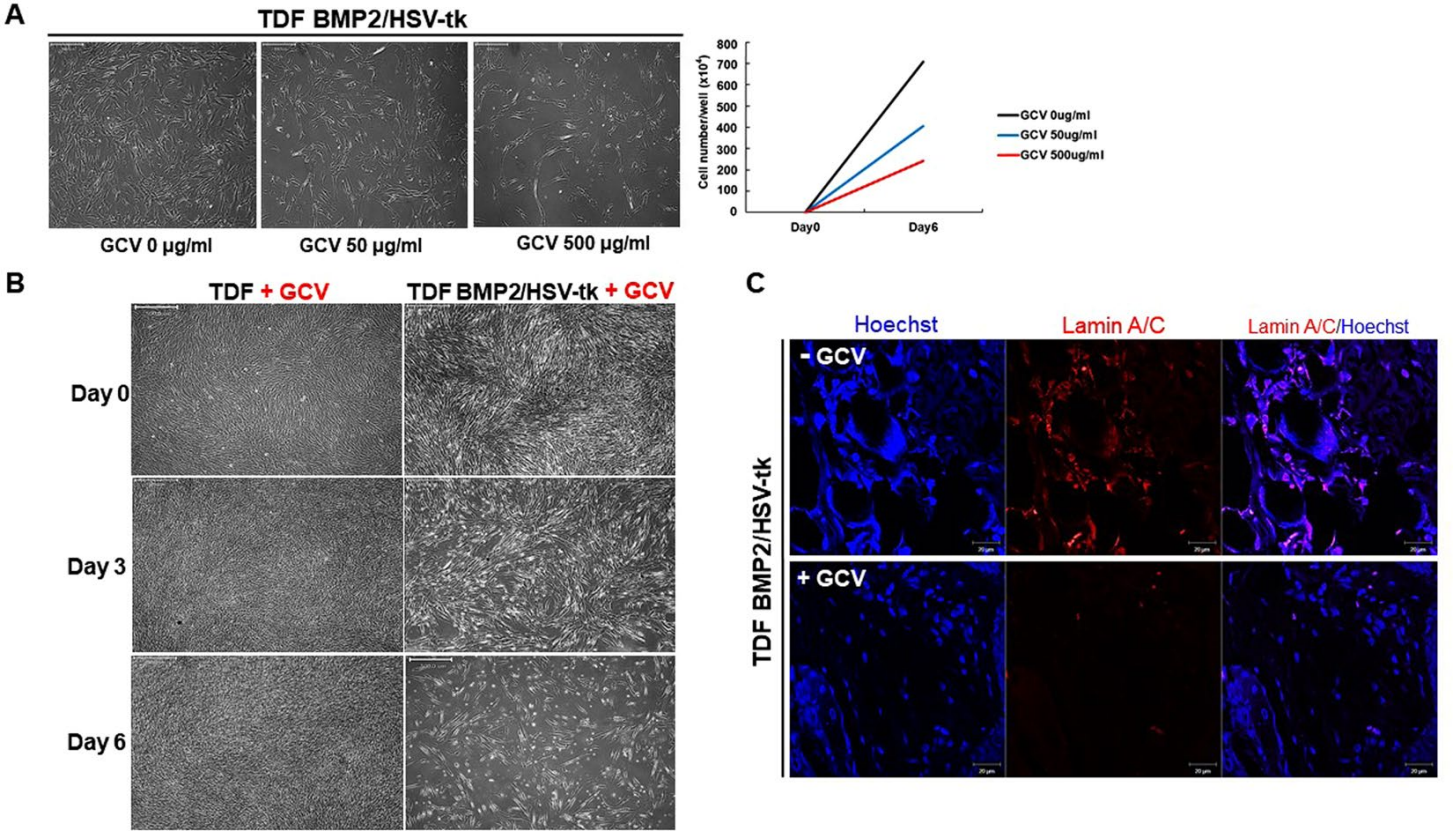
Sensitivity of TDF BMP2/HSV-tk to GCV. (**A**) TDF vehicle control and TDF BMP2/HSV-tk cells were seeded in a 24-well plate and cultured in growth medium. Next day, GCV was added at a different concentration range (0, 50, and 500 μg/mL) and cultured for 6 days. The surviving cells were counted by trypan blue staining and the cell survival rate was determined for each group. The scale bars represent 500 μm. (**B**) TDF vehicle control and TDF BMP2/HSV-tk cells were seeded in a 24-well plate and cultured in osteogenic induction medium. Next day, GCV was added at 500 μg/mL concentration and then cultured for 6 days. Representative images were visualized at days 0, 3, and 6 after treatment with GCV. Scale bars represent 500 μm. (**C**) Lamin A/C staining of subcutaneous transplanted with TDFs or TDF BMP2/HSV-tk cells after GCV treatment for 5 days. Nuclei (blue; Hoechst) and human-specific Lamin A/C (red).

We next examined whether GCV treatment can induce the apoptosis of TDF BMP2/HSV-tk cells via the expression of HSV-tk gene. The TDF vehicle control and TDF BMP2/HSV-tk cells were cultured in growth medium with or without GCV for 6 days. After exposure to GCV in TDF BMP2/HSV-tk cells, the attached cells were gradually decreased in a dose-dependent manner, but not in the TDF vehicle control. Moreover, GCV treatment at 500 μg/mL concentration significantly promoted cell death via the activation of HSV-tk gene in TDF BMP2/HSV-tk cells (Fig. 4A). TDF BMP2/HSV-tk cells were then exposed to the 500 μg/mL of GCV during *in vitro* osteogenesis. As expected, the number of TDF BMP2/HSV-tk cells were decreased upon GCV treatment at day 6 during osteogenesis (Fig. 4B), suggesting the direct cytotoxicity of GCV in HSV-tk encoding gene expressed cells. Results of *in vivo* experiment also showed that GCV treatment markedly decreased the expression levels of human-specific Lamin A/C in TDF BMP2/HSV-tk cells (Fig. 4C).

### TDF BMP2/HSV-tk form calcified bone tissue *in vivo*

**Figure 5:**
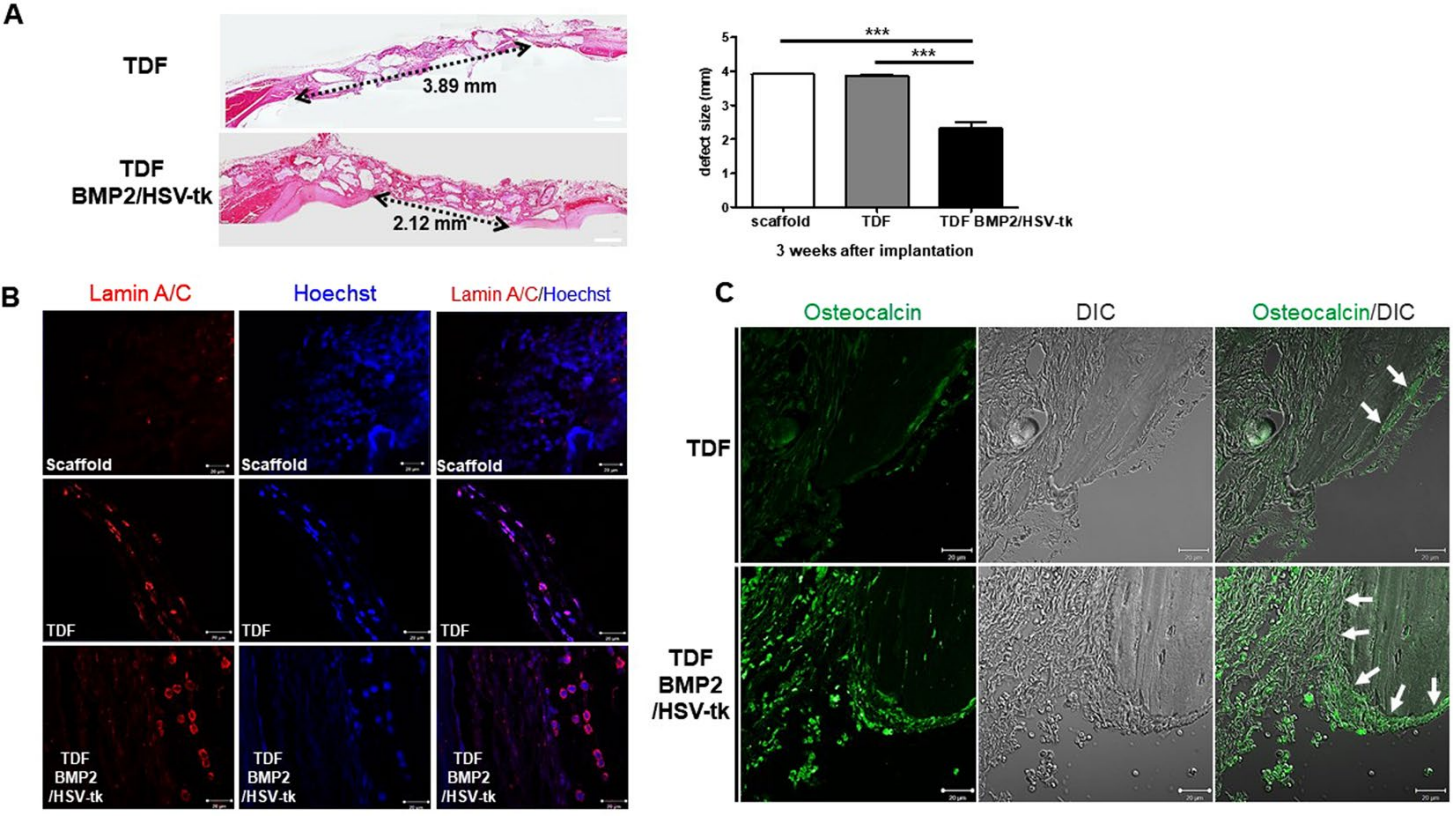
*In vivo* bone-forming ability of TDF BMP2/HSV-tk cells in a mouse cranial defect model. (**A**) (n = 4 mice/group and 2 images/mouse) H&E staining of cranial bone defects transplanted with TDFs or TDF BMP2/HSV-tk cells after 3 weeks implantation. Scale bars represent 1 mm. Bone defect size from scaffold only (data not shown), TDFs, and TDF BMP2/HSV-tk cells groups, respectively. The center of diameter for the defect region was determined from the H&E staining images. Representative image of cranial bone defects transplanted with TDFs or TDF BMP2/HSV-tk cells after 3 weeks implantation. (**B**) Nuclei (blue; Hoechst), and human-specific Lamin A/C (red) **(C)** differential interference contrast (DIC), and osteocalcin (green). Arrows: edge of defect regions. Error bars indicated mean ± SD; ***p < 0.001 compared with the corresponding control.

**Figure 6:**
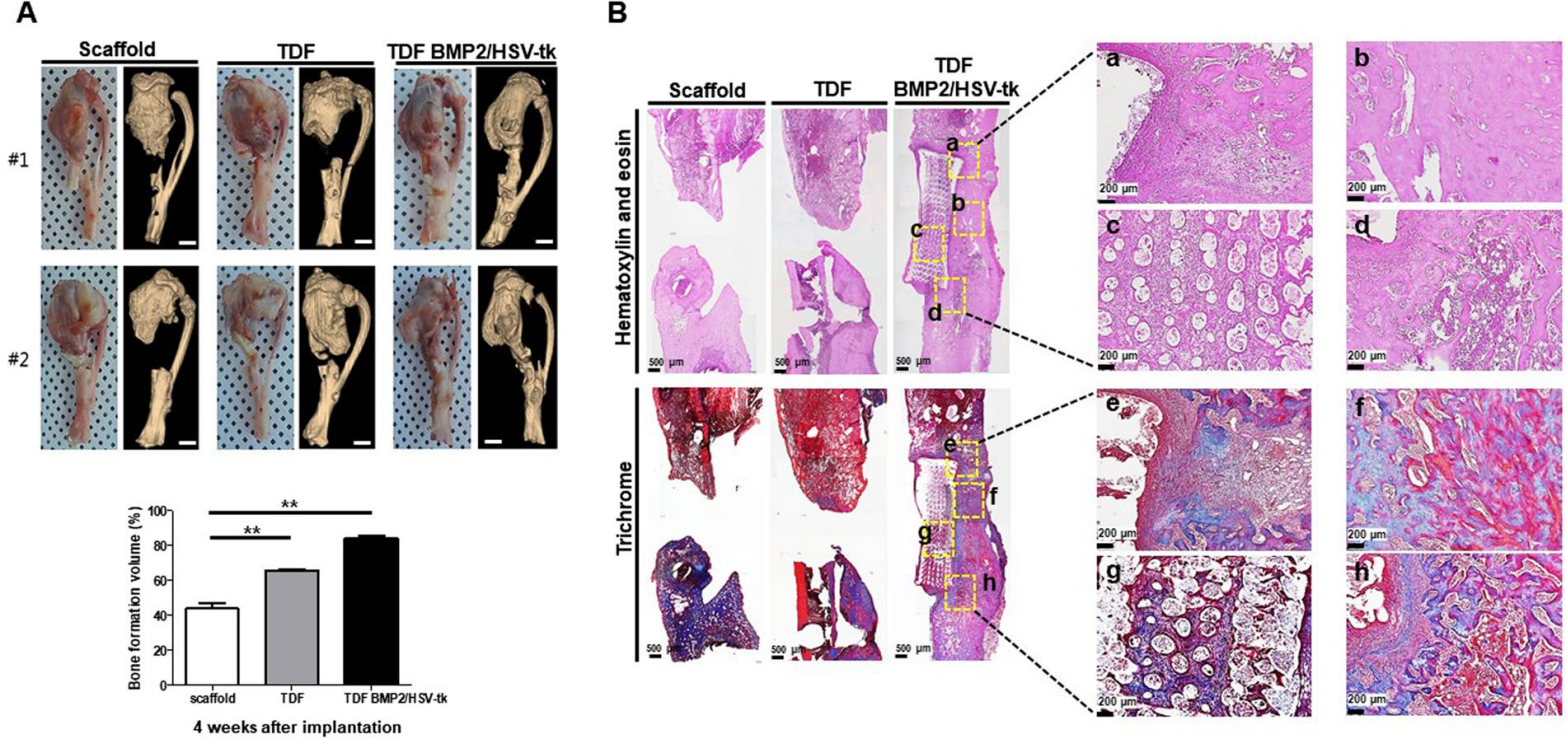
*In vivo* bone-forming ability of TDF BMP2/HSV-tk cells in a rat tibia defect model. (**A**) (n = 4 rat/group and 6 images/rat) Representative three-dimensional μCT images of tibial bone defects with scaffold only, TDF vehicle control cells, and TDF BMP2/HSV-tk at 4 weeks after implantation. Scale bars represent 500 μm. The bone formation volume of tibial bone defects in scaffold only, TDF vehicle control cells, and TDF BMP2/HSV-tk was analyzed using a bone imaging analysis software. Data were represented as mean ± SD; **p < 0.01 compared with the corresponding control. (**B**) (n = 4 rat/group and 3 images/rat) H&E staining images of tibial bone defects in scaffold only, TDF vehicle control cells, and TDF BMP2/HSV-tk at 4 weeks after implantation. Scale bars represent 200 μm. Masson’s trichrome images of tibial bone defects with scaffold only, TDF vehicle control cells, and TDF BMP2/HSV-tk at 4 weeks after implantation. Scale bars represent 200 μm. High magnification images show the mineralized bone tissue (red) in defect regions.

To determine the bone-forming ability of self-produced BMP2 in TDF BMP2/HSV-tk cells, TDF BMP2/HSV-tk cells were incubated with a scaffold in osteogenic induction medium for 24 h and then implanted into the cranial and tibia defect regions. Histological examination of the cranial tissues showed that the critical defect size (4 mm) significantly decreased in the TDF BMP2/HSV-tk group, compared with the TDF vehicle control (Fig. 5A). Next, we evaluated whether the transplanted TDF BMP2/HSV-tk cells directly contributed to the regeneration of bone tissue. Immunofluorescence staining using human-specific Lamin A/C and osteocalcin was performed for the new bone tissues with TDF BMP2/HSV-tk cells at 3 weeks after implantation. The group of transplanted TDF BMP2/HSV-tk cells clearly showed a positive signal for human-specific Lamin A/C and osteocalcin, in contrast with the TDF vehicle control and scaffold groups (Fig. 5B,C). Another examination of bone tissue repair using critical-sized tibia defect model also showed the great potential of TDF BMP2/HSV-tk cells; intense calcification appeared in the TDF BMP2/HSV-tk transplanted group during 2–3 weeks after implantation, as revealed by x-ray images (Supplementary Fig. S4A). Three dimensional micro-CT measured the bone formation volume at 4 weeks after implantation, showing significantly higher bone tissue formation in TDF BMP2/HSV-tk transplanted group compared to control groups (Supplementary Fig. S4B and Fig. 6A). In addition, we observed that the TDF vehicle control also induced new bone tissue formation, revealing the potential ability of TDF cells for bone tissue regeneration *in vivo* (Fig. 6A). H&E and trichrome staining showed the presence of new bone tissue and mineralized osteoid (blue or red) within the pores of the polycaprolactone (PCL) scaffold was remarkably observed in TDF BMP2/HSV-tk transplanted group, in contrast with the control groups (Fig. 6B). Collectively, these results indicated that the transplanted human TDF BMP2/HSV-tk cells infiltrated into the defect sites and stimulated the formation of native bone tissue.

## Discussion

Bone defects are commonly caused by tumors, infection, extensive traumas, and osteoporosis-related fractures^7^. Small bone defects spontaneously repair themselves according to a bone healing process that includes inflammation, repair, and remodeling, but the reconstruction of large bone defects can be challenging^8^. In order to heal and restore the large bone defects, several options are currently suggested as a part of the recent strategies for bone tissue engineering, mainly (1) bone graft substitutes (scaffolds), (2) healing stimulators (e.g., BMP2 or vascular endothelial growth factor (VEGF)), and (3) suitable progenitor cells (osteoblasts such as MSCs)^9^. As for the cellular candidates, MSCs are a well known cell source for increasing osteo-induction and osteogenesis, although the underlying mechanisms of MSC function in bone regeneration are still unclear^10,11^. In addition, many researchers attempted molecular engineering of MSC for improving the osteo-inductive function. Izadpanah *et al*. (2008) developed and introduced the gene delivery approaches of MSCs on bone regeneration^12^. However, MSC gene delivery is not a feasible approach, as it typically uses a virus system (lentiviral or adenoviral vectors). The application of viral methods to incorporate the desired gene into MSCs has safety issues, such as immunological and toxicological response^13^. Here, we found that TDFs have osteogenic potential as osteoblast progenitor cells, and therefore, can be used in bone tissue regeneration. Furthermore, TDFs were modified by the insertion of BMP2 and HSV-tk encoding genes using a non-viral method, which successfully confirmed both their safety and efficiency during *in vitro* and *in vivo* osteogenesis.

TDFs can be generated by patient-specific iPSC. Since the first report by Yamanaka in 2006, somatic fibroblasts have been known to be capable of reprogramming into iPSCs through the retroviral transduction of *Oct4*, *Sox2*, *Klf4*, and *c-Myc* genes^14^. The differentiation potential of iPSCs into tissues from all three germ layers has been evaluated by teratoma formation assay. Many researchers considered teratoma formation a gold-standard method to evaluate the ability of pluripotent cells^14–16^. However, we found another application of the teratoma formed by iPSCs in this study. We focused on the fibroblasts derived from teratoma, which morphologically resemble MSCs and differentiate into mesenchymal tissues, especially bone tissue. Our results suggest a possible application, i.e., TDFs can form the desired patient-specific osteoblasts if TDFs are explanted from the teratoma formed by patient-specific induced pluripotent stem cells. TDFs have a great potential as an unlimited cell source from patient iPSCs, not only for differentiating into mesenchymal tissues but also being genetically matched with the patient for immunological safety in clinical applications.

In bone research, immortalized osteoblast cell lines are used for testing novel drugs and biomaterials in *in vitro* osteogenesis as preclinical models. Most immortalized osteoblast cell lines show ease of culturing and proliferation, but their osteogenic potential was relatively low compared with the MSCs and TDFs in our study (Fig. 2B). Moreover, MG63 cell lines showed a lower ALP activity and calcium content than G292 cells, although all these cell lines were from human osteosarcoma. One of the main issues of immortalized osteoblast cell line is their unavailability for clinical study, in addition to the difference in osteogenic ability, which can affect the results of drug and biomaterial testing. However, TDFs could show a stable maintenance without phenotypic changes and easily manipulate the desired genes, whereas MSCs are very difficult to culture for both *in vitro* growth and genetic engineering. Overall, TDFs are an attractive cell source for deriving osteoblast cells towards orthopedic applications.

An ultimate goal of our study is the generation of safe and functional cell types for therapy. In terms of safety, we applied the combination of GCV and HSV-tk gene therapy system in TDF cells to eliminate the manipulated cells after cell therapy. As genetically engineered cells can cause the development of cancer, unexpected proliferation, or differentiation of native cells, we incorporated HSV-tk as a suicide gene. It has been known that cells expressing HSV-tk suicide gene trigger the phosphorylation of GCV, which leads to programmed cell death^17,18^. We successfully confirmed the kill switch system using HSV-tk gene in combination of GCV, resulting the selective elimination of HSV-tk-expressing cells by apoptosis; over 80% of such cells were removed in our present study (Fig. 4). *Hypoxanthine-guanine phosphoribosyl transferase (HGPRT)* encoding gene is located on the X chromosome and plays an important role in the generation of purine nucleotides for DNA synthesis via salvage pathway^19,20^. A cell deficient HGPRT is known to die in the presence of aminopterin due to the inhibitory effect of aminopterin on DNA *de novo* synthesis pathway^21,22^. We tried editing the *HGPRT* gene using the clustered regularly interspaced short palindromic repeats (CRISPR)-CRISPR-associated system (Cas) system in TDFs, and determined the cell death by treatment of aminopterin (Supplementary Fig. S5). These results suggested that knocking out the *HGPRT* gene expressed in TDFs could be another kill switch system, with exogenous treatment of aminopterin.

In conclusion, our study determined the osteogenic potential of TDFs originating from human pluripotent cells. In addition, BMP-2 and HSV-tk, when co-expressed in TDF cells, that could promote bone growth and healing along with controlled stem cell survival. Therefore, our study suggests that TDFs can be a good source of patient-specific osteoblasts in the clinical research on bone regeneration.

## Materials and Methods

The TDFs were generated as described elsewhere^23^. Briefly, two to three million hESCs (H9 cells) were suspended in the knockout medium, washed once with PBS, and harvested. The cell pellet was re-suspended in 30% Matrigel (Gibco, New York City, NY, USA) and injected subcutaneously into SCID mice. Six weeks after implantation, the teratoma were surgically isolated and washed with PBS (Supplementary Fig. S6A). The masses were chopped and minced into tiny pieces using a scalpel and cultured in DMEM complemented with 10% FBS, 1% non-essential amino acids, 1% penicillin-streptomycin, 2 mM glutamax, and 55uM beta-mercaptoethanol for the preparation of TDFs. All the tissue clumps were transferred to a new culture dish and incubated overnight. Fibroblast-like cells attached to the plate and the culture was incubated until the TDF cells spread out from the bottom of the tissue clumps. After 7–14 days, all viable fibroblasts from teratoma tissue fragments were separated and the remaining tissue fragments along with medium were discarded. The cultures were allowed to grow through two passages before freezing. To characterize human fibroblasts from teratomas, mRNA expression of fibroblast marker genes such as FSP-1(fibroblast specific protein-1), vimentin, and CD90 were determined by quantitative real-time PCR (Supplementary Fig. S6B). All animal works were carried out in accordance with the guidelines and regulations of the Korea University Animal Care Committee. The protocol was approved by the Korea University of Institutional Animal Care and Use Committee (IACUC).

### Cell Culture and ***in vitro*** Osteogenic Differentiation

Bone marrow human MSCs were obtained from Lonza (catalog PT-2501). MSCs and TDF cells were maintained in high glucose DMEM (Lonza, Walkersville, MD, USA) with 10% fetal bovine serum (FBS) (Gibco), 1% non-essential amino acid (NEAA) (Gibco), 1% glutamax (Gibco), 0.1% beta-mercaptoethanol (Sigma Aldrich, St. Louis, MO, USA), and 1% penicillin/streptomycin (Gibco). For the cell viability assay, MSCs and TDF cells were seeded (1 × 10^4^ cells/well) in the growth medium, and the proliferation rate was measured using the cell counting kit 8 (CCK-8) (Dojindo Laboratories, Kumamoto, Japan), following the manufacturer’s protocol. Human MG-63 and G292 cells were obtained from Korean cell line bank and cultured in DMEM supplemented with 10% FBS and 1% antibiotics (penicillin/streptomycin). NHDFs were also obtained from Korean cell line bank and maintained in RPMI (Lonza) with 25 mM Hepes, 15% FBS, and 1% penicillin/streptomycin. All the cells were incubated at 37 °C with 5% CO_2_ in their growth medium.

To induce osteogenic differentiation, the cells were seeded in 24-well plates and grown upon 90% confluency. The medium was then replaced with osteogenic induction medium (OIM) containing 10 nM dexamethasone (Sigma), 0.2 mM ascorbic acid (Sigma), and 10 mM β-glycerol phosphate (Sigma). Thereafter, the medium was changed every 3 days. The cells cultured in the growth medium served as the control.

### ALP Activity

ALP activity was measured using the colorimetric SensoLyte^®^*p*NPP Alkaline Phosphatase Assay Kit (Anaspec, Fremont, CA, USA), according to the supplier’s protocol. The cells were washed with PBS and lysed in lysis buffer containing 0.5% Triton X-100 (Sigma) on day 3 or 7 after being cultured in the osteogenic induction medium. Samples containing equal concentrations of proteins were used to determine the ALP activity, measured as the absorbance at 405 nm with a microplate reader.

### Quantitative Reverse Transcription PCR assay

Total RNA was isolated using TRIzol reagent (Invitrogen, Carlsbad, CA, USA) to analyze gene expression in human cell lines. cDNA was synthesized from 1 μg RNA using a PrimeScript™ 1^st^ strand cDNA synthesis kit (Takara Bio, Tokyo, Japan), according to the manufacturer’s instructions. Quantitative real-time polymerase chain reaction (qRT-PCR) was performed using cDNA and SYBR green dye (Applied Biosystems, Foster City, CA, USA) in ABI Prism 7300 Detection System (Applied Biosystems). Relative mRNA expression was analyzed using 2^(−∆∆Ct)^ method and was normalized with that of the gene encoding glyceraldehyde-3-phosphate dehydrogenase (*GAPDH*). The specific sequences of the primer pairs are shown in the supplemental Experimental Procedures.

### Calcium Assay and Alizarin Red S Staining

For the calcium assay, cells were washed with PBS and decalcified using 0.6 N HCl. Calcium concentration in the supernatant was measured using QuantiChrom™ Calcium Assay Kit (DICA-500; BioAssay Systems, Hayward, CA, USA), according to the manufacturer’s instructions. For Alizarin Red S staining, the cells were washed with PBS and fixed in 4% paraformaldehyde for 20 min. The cells were then washed twice with distilled water and stained with Alizarin Red S solution (Millipore, Darmstadt, Germany) for 20 min at room temperature. Finally, the cells were washed three times with 1 mL distilled water, and the color change was evaluated using photographs. ECM mineralization was quantified by adding 1 mL of 100 mM cetylpyridinium chloride (Sigma) to each well and measuring the subsequent optical density at 570 nm.

### Determination of Cell Sensitivity to GCV

Cells were plated in a 24-well plate with 10000 cells/well. Next day, GCV was added into each well at various concentrations, ranging from 0 to 500 μg/mL. Six days later, the cell survival rate was determined using trypan blue staining. The number of surviving cells was counted for each group.

For *in vivo* experiment, twelve BALB/c nude mice were divided into 2 groups with or without GCV treatment, 6 mice in each group. Each nude mouse was injected sc on both sides of flanks with 1 × 10^6^ TDFs or TDF BMP2/HSVtk cells. Next day, GCV (100 mg/kg) was given intra-peritoneal cavity (i.p), injection volume 0.2 ml/mouse, once one day, continuous injection for 5 days. After 5 days, the defective sites were analyzed with immune-histo-fluorescence staining with antibodies against lamin A/C (1:100, rabbit; Abcam, Cambridge, MA, USA). All animal study protocols were approved by the IACUC of Korea University and followed the guidelines of the Animal Care and Use Committees of Korea University.

### Bone Regeneration in the Cranial and Tibia Defect Model

All animal study protocols were approved by the IACUC of Korea University. All animals were housed in a specific pathogen-free (SPF) animal facility and followed the guidelines of the Animal Care and Use Committees of Korea University. For cranial defect model, twelve immunodeficient mice (Six-week-old female BALB/c nude mice) were anesthetized, and skin incisions were made on their parietal skull bones. A circular (4-mm diameter) critical defect was created using a trephine bur in the mice cranium. The full thickness of the cranial bone was removed and biphasic calcium phosphate (BCP) scaffold with or without cells was immediately placed in the defect. TDF and TDF/BMP2-tk laden BCP scaffolds were cultured for 1 day *in vitro* and then implanted into the defect regions. The skin of the parietal skull bones was sutured. After 3 weeks, the defective sites were analyzed with H&E staining and immune-histo-fluorescence staining.

For a tibia defect model, eight-week-old Sprague-Dawley rats were used. They were anesthetized with tiletamine/zolazepam (50 mg/kg; Zoletil) and xylazine (10 mg/kg; Rompun). After shaving the right tibia, the periosteum and soft tissue were carefully retracted and two 0.9 mm K-wires (Zimmer, Warsaw, IN) were fixed to the right tibia. A 7-mm tibia critical defect was created with a cutting burr. TDF and TDF/BMP2-tk laden polycaprolactone (PCL) scaffolds were pre-cultured for 1 day *in vitro* and then implanted into the defect regions. The subcutaneous tissue and skin were sutured. After 4 weeks, bone regeneration in the defective sites was analyzed by microcomputed tomography (μCT) system (Albira II Imaging System, Carestream Health, a voltage of 40 kV and a current of 250 μA was used with a nominal resolution of 9 μm/pixel) and histological study (H&E and Masson’s trichrome staining).

### Histochemical Analysis and Immune-histo-fluorescence Staining

At sacrifice, the defective sites were removed and fixed in 10% buffered formalin, embedded in paraffin. The paraffin embedded tissue samples were sliced into 5-μm thickness using a rotary microtome (Leica, RM2255). The tissue section was deparaffinized and dehydrated, followed by H&E, trichrome, and/or immune-histo-fluorescence staining. For H&E staining, the rehydrated sections were incubated in hematoxylin solution (Sigma) for 5 min and washed with tap water; they were then immersed in an Eosin-Y solution (Sigma) for 1 min and washed with tap water. For trichrome staining, the sections were stained with hematoxylin solution for 15 min, and then washed in acetic acid (1%) and placed in acid orange G solution. After washing, the sections were stained with light blue for 5 min and images were taken by H-filter in color mode. For immune-histo-fluorescence staining, the rehydrated sections were permeabilized in 0.1% (v/v) Triton-X in PBS at RT for 5 min and then added with the blocking solution of 10% FBS in PBS at RT for 60 min. The sections were incubated with primary antibodies against lamin A/C (1:100, rabbit; Abcam) and osteocalcin (1:100, mouse; Life technologies, Carlsbad, CA, USA) in the blocking buffer at 4 °C for overnight and washed with PBS. Next day, the sections were incubated with secondary antibodies raised against mouse (1:200, goat anti mouse; Life Technologies, Alexa Fluor 568 and 1:500, goat anti mouse; Life Technologies, Alexa Fluor 488) in the blocking solution at 4 °C for overnight and washed with PBS. The stained images were captured by confocal laser scanning microscopy (LSM700, Zeiss, Oberkochen, Germany).

### Statistical analysis

All the assays were repeated at least in triplicate (n = 3), and the representative data are expressed as mean ± standard deviation (SD). The Student’s two-tailed t-test was used to compare two groups and One-way ANOVA was used to determine multiple groups at the same time point by SPSS software. A *P* values less than 0.05 was considered statistically significant.

## Electronic supplementary material

supplementary information

## References

[1] Tocci A, Forte L (2003). Mesenchymal stem cell: use and perspectives. The hematology journal: the official journal of the European Haematology Association.

[2] Kim HJ, Park J-S (2017). Usage of Human Mesenchymal Stem Cells in Cell-based Therapy: Advantages and Disadvantages. Development & Reproduction.

[3] Richardson SM (2010). Mesenchymal stem cells in regenerative medicine: opportunities and challenges for articular cartilage and intervertebral disc tissue engineering. Journal of cellular physiology.

[4] Ozolek, J. A. & Castro, C. A. in *Embryonic Stem Cells - Basic Biology to Bioengineering* (ed Michael S. Kallos) Ch. 13 (InTech, 2011).

[5] Gropp M (2012). Standardization of the teratoma assay for analysis of pluripotency of human ES cells and biosafety of their differentiated progeny. PloS one.

[6] Cho SM, Park JS, Min B, Kwon S, Kang YK (2015). Rapid generation of secondary fibroblasts through teratoma formation. BioTechniques.

[7] Frohlich M (2008). Tissue engineered bone grafts: biological requirements, tissue culture and clinical relevance. Current stem cell research & therapy.

[8] Eslaminejad, M. B. & Faghihi, F. in *Regenerative Medicine and Tissue Engineering - Cells and Biomaterials* (ed Daniel Eberli) Ch. 03 (InTech, 2011).

[9] Oryan A, Kamali A, Moshiri A, Baghaban Eslaminejad M (2017). Role of Mesenchymal Stem Cells in Bone Regenerative Medicine: What Is the Evidence. Cells, tissues, organs.

[10] Li H (2007). Bone regeneration by implantation of adipose-derived stromal cells expressing BMP-2. Biochemical and biophysical research communications.

[11] Hayashi O, Katsube Y, Hirose M, Ohgushi H, Ito H (2008). Comparison of osteogenic ability of rat mesenchymal stem cells from bone marrow, periosteum, and adipose tissue. Calcified tissue international.

[12] Izadpanah R, Bunnell BA (2008). Gene delivery to mesenchymal stem cells. Methods in molecular biology (Clifton, N.J.).

[13] Oggu GS (2017). Gene Delivery Approaches for Mesenchymal Stem Cell Therapy: Strategies to Increase Efficiency and Specificity. Stem cell reviews.

[14] Takahashi K, Yamanaka S (2006). Induction of pluripotent stem cells from mouse embryonic and adult fibroblast cultures by defined factors. Cell.

[15] Nelakanti RV, Kooreman NG, Wu JC (2015). Teratoma Formation: A Tool for Monitoring Pluripotency in Stem Cell Research. Current protocols in stem cell biology.

[16] Gutierrez-Aranda I (2010). Human Induced Pluripotent Stem Cells Develop Teratoma More Efficiently and Faster Than Human Embryonic Stem Cells Regardless the Site of Injection. Stem Cells (Dayton, Ohio).

[17] Wang J, Lu X-X, Chen D-Z, Li S-F, Zhang L-S (2004). Herpes simplex virus thymidine kinase and ganciclovir suicide gene therapy for human pancreatic cancer. World Journal of Gastroenterology.

[18] Beck C, Cayeux S, Lupton SD, Dorken B, Blankenstein T (1995). The thymidine kinase/ganciclovir-mediated “suicide” effect is variable in different tumor cells. Human gene therapy.

[19] Sculley DG, Dawson PA, Emmerson BT, Gordon RB (1992). A review of the molecular basis of hypoxanthine-guanine phosphoribosyltransferase (HPRT) deficiency. Human genetics.

[20] Arnold, W. J. & Kelley, W. N. in *Purine Metabolism in Man: Enzymes and Metabolic Pathways* (eds Oded Sperling, Andre De Vries, & James B. Wyngaarden) 203–209 (Springer US, 1974).

[21] Guibinga G-H, Murray F, Barron N, Pandori W, Hrustanovic G (2013). Deficiency of the purine metabolic gene HPRT dysregulates microRNA-17 family cluster and guanine-based cellular functions: a role for EPAC in Lesch-Nyhan syndrome. Human Molecular Genetics.

[22] Parker, J. in *Encyclopedia of Genetics* (ed Jefferey H. Miller) 60 (Academic Press, 2001).

[23] Song H, Chung SK, Xu Y (2010). Modeling disease in human ESCs using an efficient BAC-based homologous recombination system. Cell stem cell.

