# Supplementary material for: Engineering functional BMP-2 expressing teratoma-derived fibroblasts for enhancing osteogenesis
Source: Sci Rep. 2018 Oct 1;8:14581. doi: 10.1038/s41598-018-32946-6 (PMC6167319; doi:10.1038/s41598-018-32946-6)
Supplement: Supplementary file 1 — supplementary information [file 41598_2018_32946_MOESM1_ESM.pdf]

Engineering functional BMP-2 expressing teratoma-derived fibroblasts for enhancing osteogenesis

Yoon Young Go[1], Ji Yeon Mun[2], Sung-Won Chae[1], Shin Hye Kim[1], Hoseok Song[2]* and Jae-Jun Song[1]*

Supplemental figures and figure legends

Figure. S1. Osteogenic potential of TDFs.

(A) TDFs were cultured with or without the osteogenic induction medium. ALP activity was determined at days 3 and 7. **(B)** TDFs were cultured with osteogenic induction medium for 21 days and then the calcium deposition was compared with the TDFs cultured in growth medium (TDF GM). **(B)** The expression levels of *Runx2*, *Osteocalcin*, and *Osteopontin* were evaluated at days 0, 7, and 21 in the TDFs cultured in osteogenic induction medium by real-time PCR. **(D)** TDFs differentiated in the osteogenic induction medium for 21 days, after which, they were stained with Alizarin Red S solution. Control TDF cells were cultured in growth medium (GM). Scale bars, 500 μm. Data were represented as mean ± SD; $*p < 0.05$, $**p < 0.01$, and $***p < 0.001$ compared with the corresponding control.

Figure. S2. Generation of TDF BMP2/HSV-tk

(A) Human TDF BMP2/HSV-tk is co-expressed with an osteo-inductive factor and a suicide gene by incorporating *BMP2* and *HSV-tk* encoding genes flanking internal ribosome entry site (IRES) elements. **(B)** We transfected TDF BMP2/HSV-tk cells with *cre* plasmid to activate the *BMP2* and *HSV-tk* genes. BMP2 expression of TDF BMP2/HSV-tk was determined by ELISA, and 2 ng/mL of BMP2 was quantitatively detected in these cells at 48 h. **(C)** TDFs, MSCs, and TDF BMP2/HSV-tk were seeded in a 96-well plate and then cultured in each growth medium.

Cell viability was determined by trypan blue exclusion test during 6 days. Error bars indicated mean ± SD; $^{**}p < 0.01$ and $^{***}p < 0.001$ compared with the corresponding control.

Figure. S3. BMP2 secretion from TDF BMP2/HSV-tk during osteogenesis involves BMP2 RIB.

(A) TDF vehicle control and TDF BMP2/HSV-tk cells were cultured with or without the osteogenic induction medium for 4 days, after which, the expression levels of *BMP2* gene were determined by quantitative real-time PCR. **(B)** TDF vehicle control and TDF BMP2/HSV-tk cells were cultured in osteogenic induction medium, and then the medium was collected from each cell group at days 3 and 7. BMP2 expression was determined by ELISA assay. **(C)** TDF vehicle control and TDF BMP2/HSV-tk cells were cultured with or without osteogenic induction medium for 7 days. The expression levels of *BMP2 RIB* and *RII* receptor genes were analyzed by quantitative real-time PCR. Error bars indicated mean ± SD; $^{*}p < 0.05$, $^{**}p < 0.01$, and $^{***}p < 0.001$ compared with the corresponding control.

Figure. S4. Representative x-ray images of critical-defected bone with healing.

(A) X-ray images according to three groups (scaffold, scaffold + TDF, and scaffold + TDF BMP2/HSV-tk) were presented here. Healing process of tibial bone defects in three groups was analyzed every week for 4 weeks. **(B)** The bone formation volume of tibial bone defects in defect only, scaffold, TDF, MSC, TDF+rhBMP2, and TDF BMP2/HSV-tk was analyzed using

a bone imaging analysis software. Data were represented as mean ± SD; *$p < 0.05$, **$p < 0.01$, and ***$p < 0.001$ compared with the corresponding control.

Figure. S5. Representative images of TDF BMP2/HSV-tk and TDF BMP2/HSV-tk/HGPRT KO within aminopterin medium.

TDF BMP2/HSV-tk and TDF BMP2/HSV-tk/HGPRT KO (knock out of *HGPRT* gene in TDF BMP2/HSV-tk) cells were seeded in a 24-well plate and cultured in growth medium. Next day, HAT (hypoxanthine-aminopterin-thymidine) medium was added at a typical concentration (100uM of hypoxanthine, 0.4uM of aminopterin, and 16uM of thymidine) and cultured for 48 hours. The scale bars represent 500 μm.

Figure. S6. Comparison of TDF and normal human dermal fibroblasts (NHDF).

(A) Representative image of Teratoma after isolation from SCID mice. **(B)** The expression levels of fibroblasts marker genes encoding FSP-1, Vimentin, and CD90 were evaluated in TDFs. Human middle ear epithelium cells (HMEECs) and NHDF were used as a control. Error bars indicated mean ± SD; ***$p < 0.001$ compared with the corresponding control.

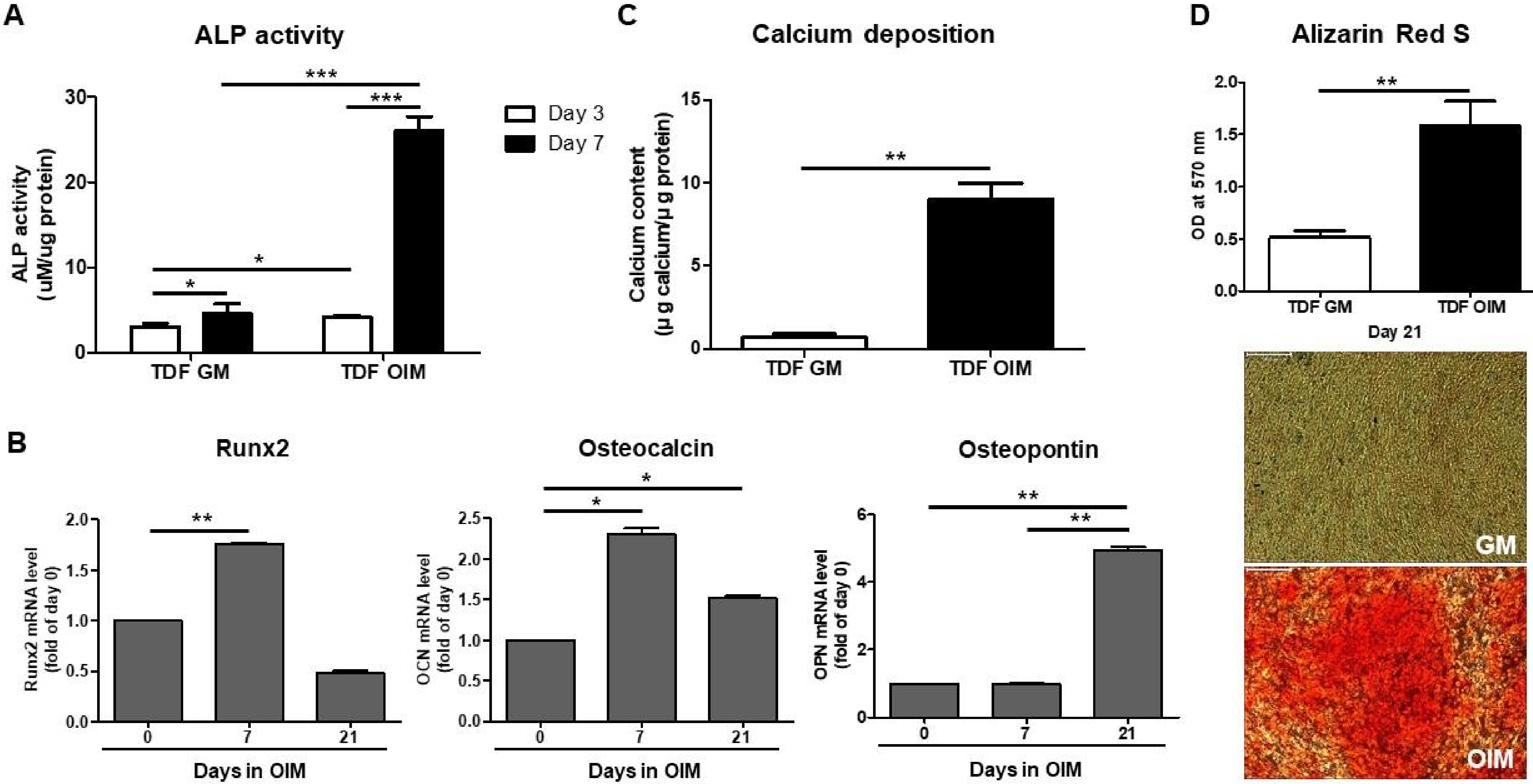
A
ALP activity
ALP activity
(uM/ug protein)
30
20
10
0
***
***
*
*
TDF GM
TDF OIM
Day 3
Day 7
C
Calcium deposition
Calcium content
(μ g calcium/μ g protein)
15
10
5
0
**
TDF GM
TDF OIM
D
Alizarin Red S
OD at 570 nm
2.0
1.5
1.0
0.5
0.0
**
TDF GM
TDF OIM
Day 21
GM
OIM
B
Runx2
Runx2 mRNA level
(fold of day 0)
2.0
1.5
1.0
0.5
0.0
**
0
7
21
Days in OIM
Osteocalcin
OCN mRNA level
(fold of day 0)
2.5
2.0
1.5
1.0
0.5
0.0
*
*
0
7
21
Days in OIM
Osteopontin
OPN mRNA level
(fold of day 0)
6
4
2
0
**
**
0
7
21
Days in OIM

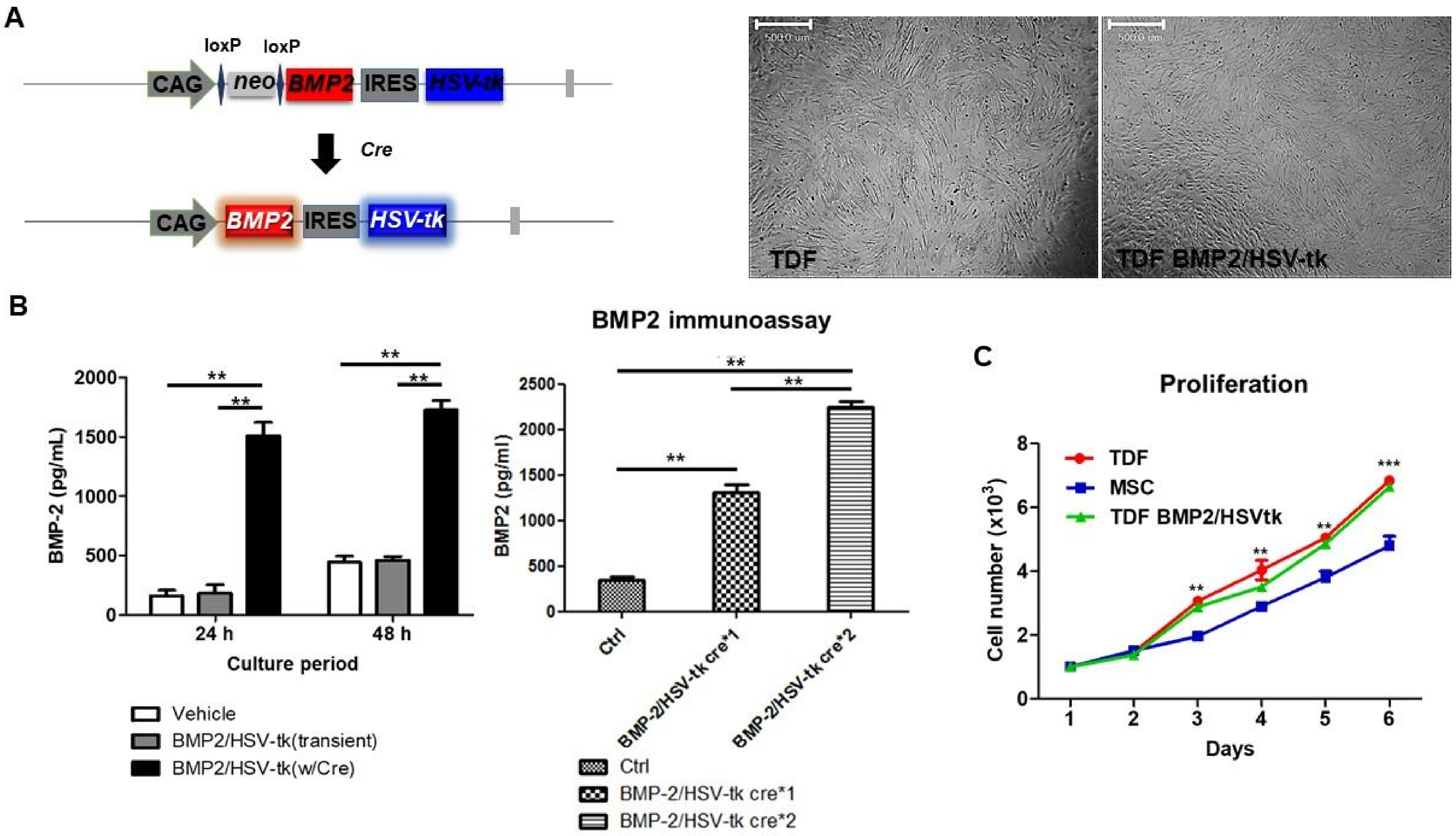
A
loxP
loxP
CAG
neo
BMP2
IRES
HSV-tk
Cre
CAG
BMP2
IRES
HSV-tk
TDF
TDF BMP2/HSV-tk
B
BMP2 immunoassay
BMP-2 (pg/mL)
24 h
48 h
Culture period
Vehicle
BMP2/HSV-tk(transient)
BMP2/HSV-tk(w/Cre)
BMP2 (pg/ml)
Ctrl
BMP-2/HSV-tk cre*1
BMP-2/HSV-tk cre*2
C
Proliferation
TDF
MSC
TDF BMP2/HSVtk
Cell number (x10³)
Days

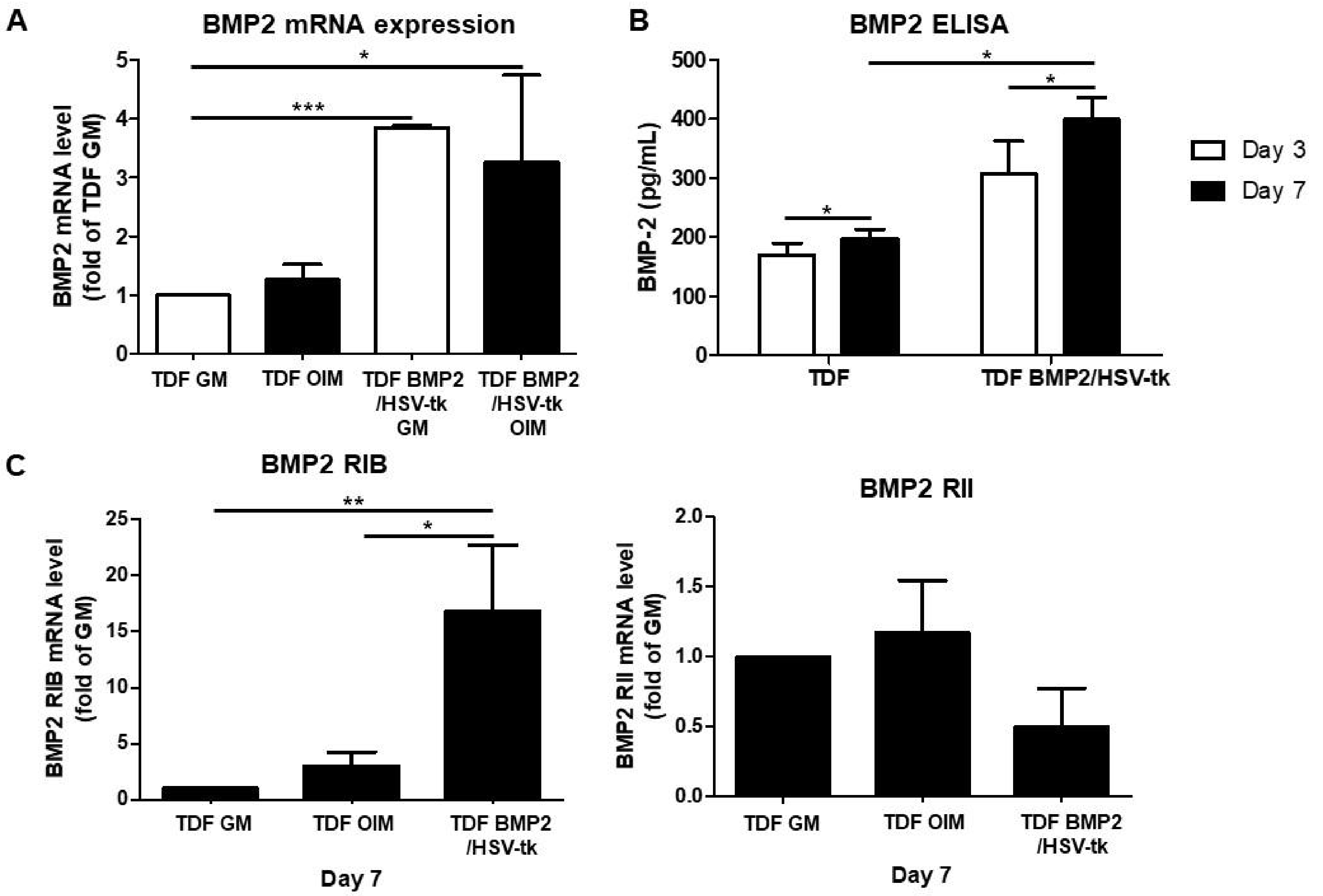

A
BMP2 mRNA expression
BMP2 mRNA level (fold of TDF GM)
TDF GM
TDF OIM
TDF BMP2 /HSV-tk GM
TDF BMP2 /HSV-tk OIM
B
BMP2 ELISA
BMP-2 (pg/mL)
TDF
TDF BMP2/HSV-tk
Day 3
Day 7
C
BMP2 RIB
BMP2 RIB mRNA level (fold of GM)
TDF GM
TDF OIM
TDF BMP2 /HSV-tk
Day 7
BMP2 RII
BMP2 RII mRNA level (fold of GM)
TDF GM
TDF OIM
TDF BMP2 /HSV-tk
Day 7

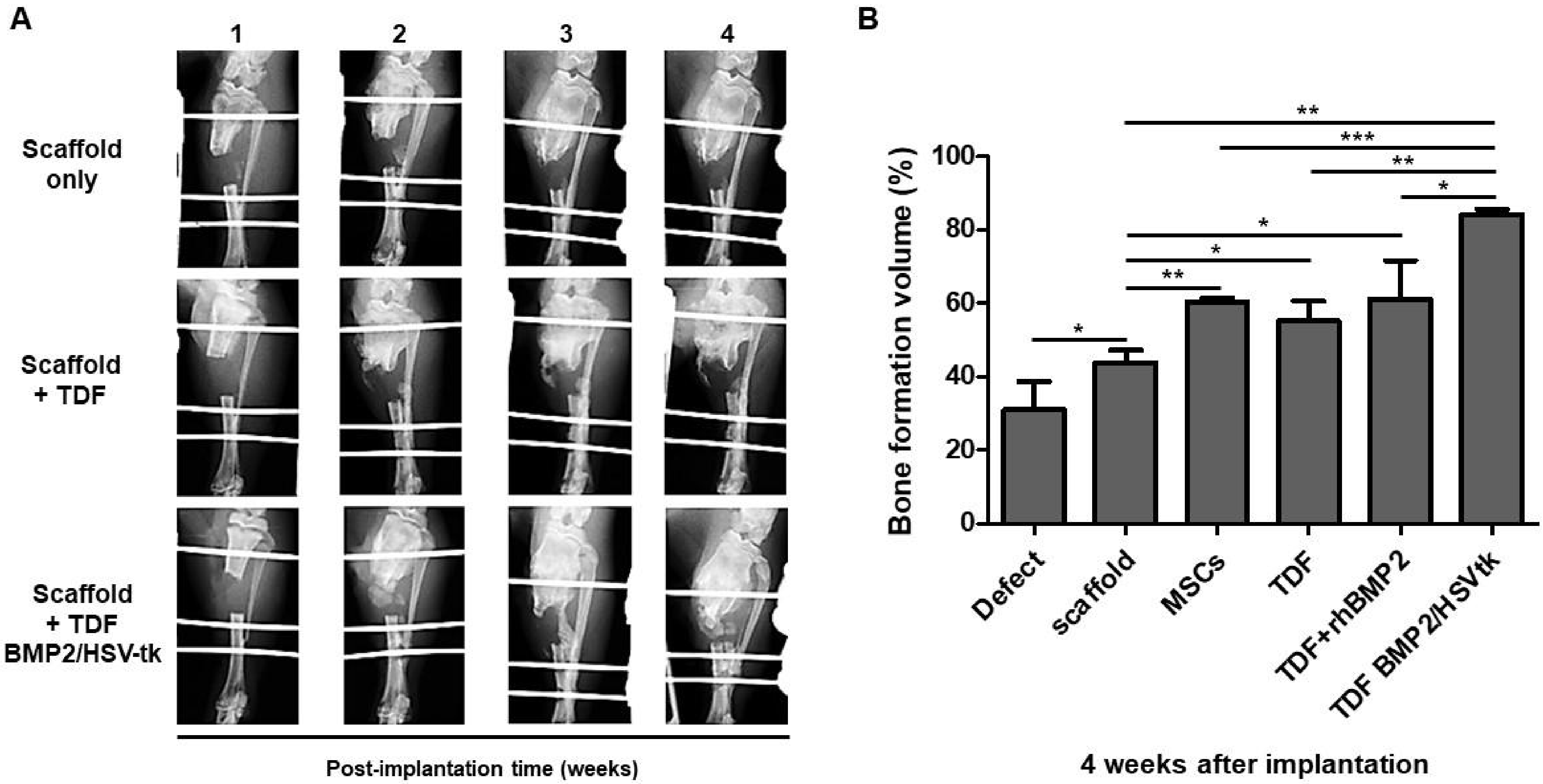
A
1
2
3
4
Scaffold only
Scaffold + TDF
Scaffold + TDF BMP2/HSV-tk
Post-implantation time (weeks)
B
Bone formation volume (%)
100
80
60
40
20
0
Defect
scaffold
MSCs
TDF
TDF+rhBMP2
TDF BMP2/HSVtk
4 weeks after implantation

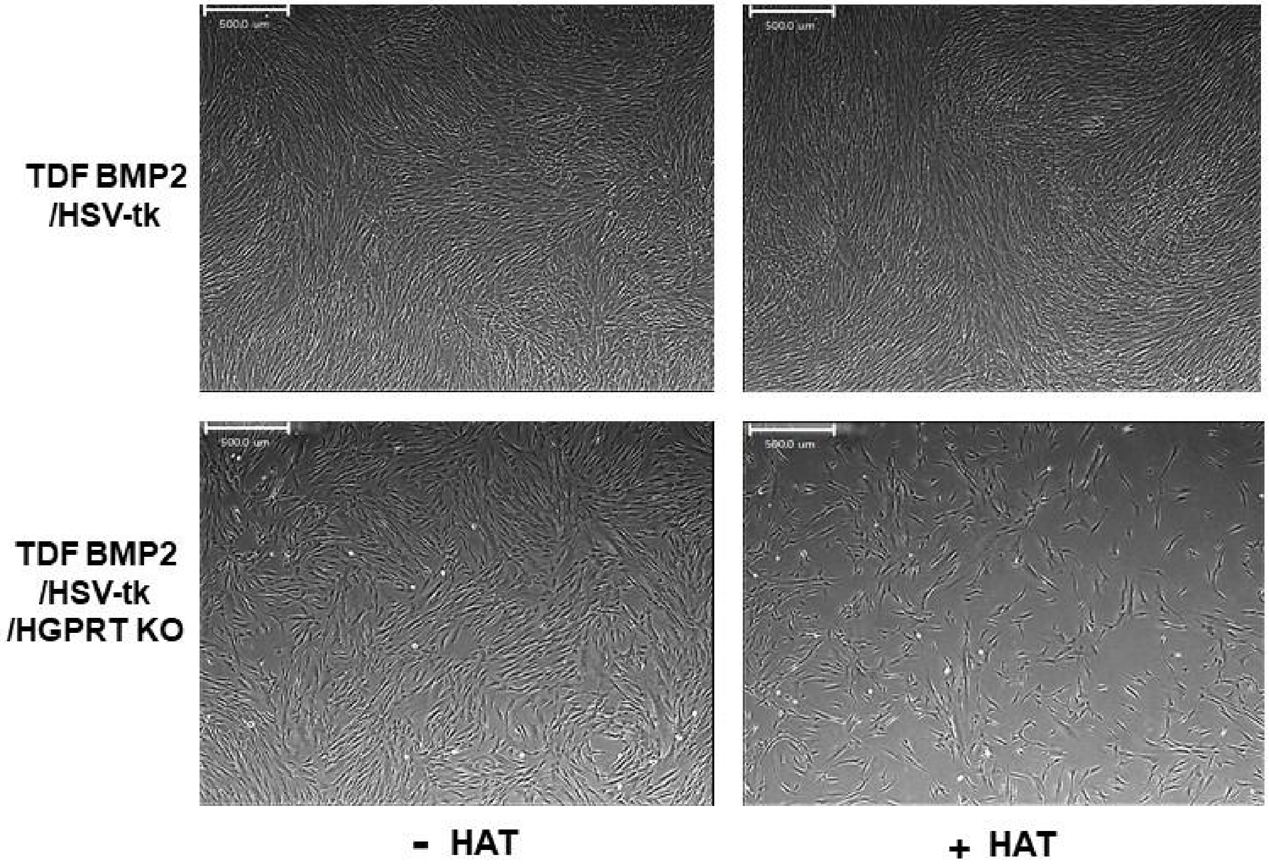
TDF BMP2
/HSV-tk
TDF BMP2
/HSV-tk
/HGPRT KO
500.0 um
500.0 um
500.0 um
500.0 um
- HAT
+ HAT

A

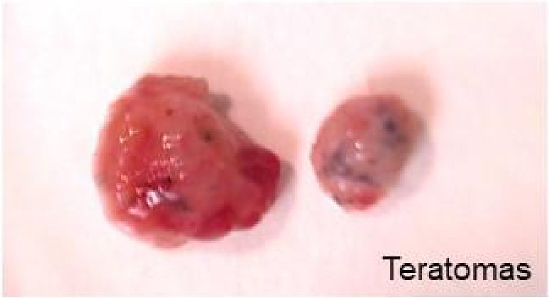
Teratomas

B

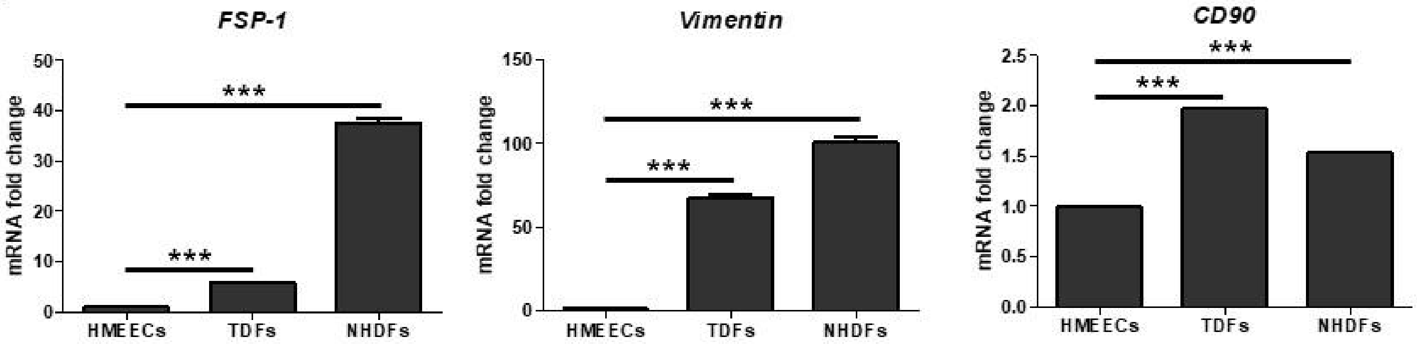
FSP-1
Vimentin
CD90
mRNA fold change
***
HMEECs
TDFs
NHDFs
50
40
30
20
10
0
150
100
50
2.5
2.0
1.5
1.0
0.5
0.0

Supplemental Experimental Procedures

Generation of stably transfected TDF BMP2/HSV-tk cell lines

The coding regions of BMP2 and HSV-tk were amplified from a human cDNA library and sub-cloned into CMV early enhance/chicken beta-actin (CAG) promoter loxP-neo-loxP plasmid. The IRES-Puro DNA fragment was inserted between BMP2 and HSV-tk, so that these two encoding genes are expressed simultaneously (Figure S2A). Construction of CAG promoter loxP-neo-loxP BMP2-IRES- HSV-tk introduced plasmid transfected into TDF cells and selected TDF BMP2/HSV-tk inactivated cells by neomycin for 2 weeks. To activate BMP2 and HSV-tk genes, *cre* plasmid was transfected into the inactivated TDF BMP2/HSV-tk cells two-three times. The expression of BMP2 and HSV-tk was confirmed by BMP2 ELISA assay and (ganciclovir) GCV treatment, respectively (Figure S2B and S2C).

Quantitative reverse transcription PCR assay

The sequence of the primers are as follows: ALP 5' TAACATCAGGGACATTGACG 3', 5' TGCTTGTATCTCGGTTTGAA 3'; IBSP 5' GAACAAGGCATAAACGGCACC 3', 5' TTCTGCATTGG CTCCAGTGAC 3'; RUNX2 5' GACACCACCAGGCCAATC 3', 5' AGAACAAGGGGGCCGTTA 3'; OSTERIX 5' GCCAGAAGCTGTGAAACCTC 3', 5' TGATGGGGTCATGGTGTCTA 3'; OCN 5' TGA CGAGTTGGCTGACCA 3', 5' GCCGTAGAAGCGCCGATAGGC 3'; OPN 5' AGGCATCACCTGTGCCATAC3', 5' GATGGGTCAGGGTTTAGCCA 3'; BMP2 5' TCAGCAGAGC TTCAGGTTTT 3', 5' AGTGACGTGGGGTGGAA 3'; BMP2 RIB 5' CATGCTTTTGCGAAGTGCAG 3', 5'

GGATGACTCTGGG TTGCCTG 3'; BMP2 RII 5' AAGCGAGGTTGGCACTATCA 3', 5' GCGGCCCTGGCGGGTGCCCTG 3'; FSP1 5' AACTTGTCACCCTCTTTGCC 3', 5' TCCTCAGCGCTTCTTCTTTC 3'; VIMENTIN 5' GCAAAGATTCCACTTTGCGT 3', 5' GAAATTGCAGGAGGAGATGC 3'; CD90 5' ATGAACCTGGCCATCAGCA 3', 5' GTGTGCTCAGGCACCCC 3'; GAPDH 5' TCGCCCCACTTGATTTTGG 3', 5' GCAAATTCCATGGCACCGT 3'

Enzyme-linked immunosorbent assay (ELISA)

BMP2 concentration in the culture medium was determined using respective ELISA immunoassay kits (PeproTech, Rocky Hill, NJ, USA). Standards and samples were incubated and pre-coated with polyclonal antibodies against human BMP2 for 2 h, after which, unbound proteins were removed by three washes. BMP2 conjugated secondary antibodies were added to the microplate wells for 2 h, and a substrate solution was added to the wells for color change. The concentration of BMP2 was measured at 450 nm using a microplate reader.
